# Cleaning, Chasing and Calming: Promising Paradigms of Senotherapy in Aging-Related Diseases

**DOI:** 10.7150/ijbs.138243

**Published:** 2026-07-20

**Authors:** Shasha Ran, Siyu Zhou, Haotian Ma, Fang Zhang, Yanrui Yang, Maiheliya Abulajiang, Xiujuan Yang, Shanshan Li, Qixiong Zhang

**Affiliations:** 1 College of Pharmacy and Food, Key Laboratory of Research and Application of Ethnic Medicine Processing and Preparation on the Qinghai-Tibet Plateau, Southwest Minzu University, Chengdu 610041, China.; 2 Department of Pharmacy, Personalized Drug Research and Therapy Key Laboratory of Sichuan Province, Sichuan Academy of Medical Science & Sichuan Provincial People's Hospital, School of Medicine, University of Electronic Science and Technology of China, Chengdu 610072, China.; 3 Department of Pharmacy, Sichuan Provincial People's Hospital East Sichuan Hospital & Dazhou First People's Hospital, Dazhou, 635000, China.

**Keywords:** cellular senescence, senolytics, immuno-senolytics, senomorphics, epigenetic regulation

## Abstract

With the acceleration of global population aging, the pathological accumulation of senescent cells (SnCs) has been confirmed as the core biological mechanism driving multiple aging-related diseases. This article aims to systematically review the formation mechanism of SnCs and their pathological roles in various tissue lesions, and to specifically evaluate the progress of three cutting-edge intervention strategies (Senolytics, Immuno-senolytics and Senomorphics). Senolytics selectively induces programmed apoptosis in SnCs by antagonizing senescent cell anti-apoptotic pathways. Immuno-senolytics include vaccines, engineered cell therapy and specific antibodies, which utilize the immune surveillance mechanism to achieve efficient elimination of SnCs. Senomorphics precisely reshape the SASP profile by targeting signaling axis or interfering with epigenetic modifications. Although the strategies have demonstrated remarkable potential for reversing pathology, their clinical translation still faces challenges such as the heterogeneity of SnCs, the lack of specific markers, and potential off-target toxicity. Future research should focus on multidisciplinary collaboration, aiming to optimize spatiotemporal targeted delivery systems and combination drug regimens to build a safer and more precise anti-senescence treatment system.

## 1. Introduction

According to the World Health Organization, the global population aged 60 years and older is projected to double from 1 billion in 2020 to 2.1 billion by 2050, while the cohort aged 80 or older will triple to approximately 426 million 1. This profound demographic shift inevitably precipitates a dramatic surge in the incidence of aging-related diseases (ARDs)—such as chronic metabolic disorders, atherosclerosis, and neurodegenerative diseases—imposing an escalating burden on global public health systems and social infrastructure 2-5. Therefore, unlocking the driving forces behind cellular aging and developing targeted therapeutic interventions have become increasingly imperative. In recent years, cellular senescence has been recognized as the core pathological mechanism driving the imbalance of tissue homeostasis and the decline of multiple organ functions. Senescent cells (SnCs) usually result from telomere depletion, persistent DNA damage response, or irreversible cell cycle arrest induced by tumor-related stress 6. SnCs exhibit high metabolic activity and acquire characteristic senescence-associated secretory phenotype (SASP). SASP not only remodel the local tissue microenvironment by releasing pro-inflammatory factors, matrix metalloproteinases (MMP) and growth factors, but also induce systemic inflammaging, thereby accelerating the pathological progression 7.

Current preclinical studies have confirmed that the pathological accumulation of SnCs in diseased tissues such as the lungs, cardiovascular system, and bone joints is a key driving force for tissue fibrosis and loss of regenerative capacity. For instance, in idiopathic pulmonary fibrosis or intervertebral disc degeneration models, SnCs activate senescent cell anti-apoptotic pathways (SCAPs) to generate apoptosis resistance and form a pro-fibrotic microenvironment locally 8-10. Based on this, developing targeted precision intervention strategies against SnCs has become the forefront of regenerative medicine and pharmacological research.

The current three cutting-edge intervention strategies of senotherapy are Senolytics, Immuno-senolytics and Senomorphics 11. Senolytics inhibiting anti-apoptotic proteins such as BCL-2/BCL-xL or interfering with the HSP90 mediated pro-survival molecular chaperone network, they accurately trigger programmed cell death of SnCs 12. Immuno-senolytics, including engineered CAR-NK/T cells, senolytic vaccines targeting GPNMB, and monoclonal antibodies, utilized the immune system to achieve long-term monitoring and targeted elimination of SnCs 13. Senomorphics are designed to reorganize the SASP profile to alleviate its paracrine damage, rather than directly mediating cell death 14. Although these emerging therapies demonstrate remarkable potential in reversing tissue damage and prolonging healthy lifespan, their transition from the laboratory to clinical application is still hindered by bottlenecks such as the high heterogeneity of SnCs, the lack of specific markers, and the systemic toxicity of long-term administration. This article aims to systematically review the generation mechanism of SnCs and their intrinsic connections with ARDs, and focuses on discussing the molecular pharmacological mechanisms, temporal and spatial targeted delivery potential, and transformation challenges of the above three types of strategies, striving to provide systematic theoretical references for the construction of the next generation of precise and safe anti-aging intervention systems.

## 2. SnCs and ARDs

### 2.1. Generation mechanism of SnCs

**Figure 1:**
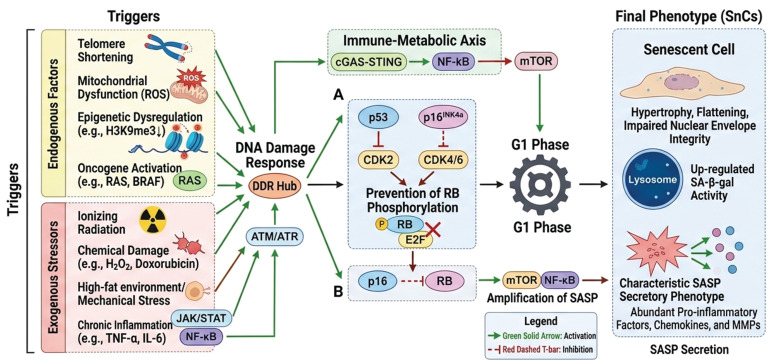
Schematic illustration of the molecular mechanisms underlying cellular senescence induction and SASP formation. Endogenous insults (telomere attrition, ROS accumulation, epigenetic dysregulation, and oncogene activation) together with exogenous stressors (irradiation, chemical damage, metabolic disturbances, and chronic inflammation) cooperatively activate the DNA damage response (DDR) and ATM/ATR signaling pathways. These signals subsequently induce permanent G_1_-phase cell-cycle arrest through the p53-p21-RB and p16^INK4a^-RB pathways by suppressing CDK activity and preventing RB phosphorylation. Concurrently, persistent activation of the cGAS-STING, NF-κB, and mTOR signaling pathways promotes SASP secretion and establishment of the characteristic senescent phenotype. Green arrows indicate activation, red arrows indicate inhibition, and black arrows represent signaling direction.

Cellular senescence refers to a stable and irreversible proliferative arrest state that cells induce when subjected to continuous stress or pathological damage (such as DNA damage, activation of oncogenes, oxidative stress, etc.) 15. As a key evolutionary protective mechanism for the body to cope with internal and external pressures, SnCs exhibit profound phenotypic reconfiguration at the biochemical and physiological levels. Their core characteristics include: morphological changes (significant cell hypertrophy, flattening, and damage to the nuclear membrane); enhanced lysosomal function (the upregulation of senescence-associated β-galactosidase (SA-β-gal) activity); and the formation of SASP, which involves the coordinated release of a large number of pro-inflammatory factors, chemokines, and MMPs 16, 17. The generation of SnCs is regulated by both endogenous depletion and exogenous stress. Multiple signaling stimuli ultimately converge on specific molecular pathways, leading to a permanent arrest of the cell cycle (**Figure 1**).

At the endogenous level, telomere shortening is the core cause of replicative senescence. As the number of divisions increases, telomere depletion triggers the ATM/ATR-p53-p21^WAF1/CIP1^ pathway, forcing the cells to exit the cell cycle 15, 18-21. Additionally, mitochondrial dysfunction generates reactive oxygen species (ROS) that cause DNA double-strand breaks, which recruit γH2AX to trigger a persistent DNA damage response 22-25. At the epigenetic level, disorders such as the reduction of H3K9me3 can promote the formation of senescence-associated heterochromatin foci, inhibiting the transcription of proliferation-related genes at the chromatin structure level 26-28. Meanwhile, the activation of oncogenes (such as RAS, BRAF) can induce oncogene-induced senescence (OIS), constructing a crucial endogenous anti-tumor barrier 29, 30.

At the exogenous level, ionizing radiation or chemical damage (such as hydrogen peroxide, chemotherapeutic drugs like doxorubicin) can directly damage the genome and induce stress-induced premature senescence 31-35. Metabolic disorders (such as a high-fat environment) and physical mechanical stress further induce aging by activating the cGAS-STING or TGF-β/Smad signaling pathways 36. In addition, factors such as TNF-α and IL-6 in the chronic inflammatory microenvironment form a positive feedback loop through the JAK/STAT or NF-κB pathways, maintaining and strengthening the aging phenotype 37, 38.

The above stress signals are ultimately executed through two core pathways: one is the p53-p21-RB pathway, which inhibits the activity of CDK2 through p21; the other is the p16^INK4a^-RB pathway, which blocks RB phosphorylation by inhibiting CDK4/6, causing the cells to permanently remain in the G1 phase 39-42. At the same time, the continuous activation of the mTOR/NF-κB axis amplifies the inflammatory effect of SASP, causing the complex pathological and physiological characteristics of SnCs 43.

### 2.2. Pathological characteristics of SnCs

**Figure 2:**
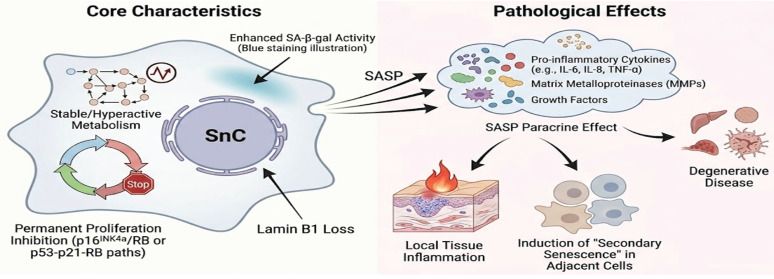
The core characteristics of SnCs include: inactive/overactive metabolism, permanent cell cycle arrest, loss of lamin B1, and increased SA-β-gal activity. SnCs exhibit a senescence associated secretory phenotype, which involves the massive release of cytokines including pro-inflammatory cytokines, MMPs, etc., leading to a series of pathological changes, including local tissue inflammation, and secondary senescence of adjacent cells through paracrine effects, ultimately resulting in degenerative disease.

The most distinctive biological feature of SnCs is the restricted proliferation under active metabolism. Although permanent proliferation inhibition has been achieved through the p16^INK4a^/RB or p53-p21-RB pathways, the metabolic network of SnCs remains stable or even hyperactive 44, 45. Besides the classic characteristics such as enhanced SA-β-gal activity and loss of nuclear lamina protein Lamin B1, the pathological driving force of SnCs mainly stems from the paracrine effect of SASP (**Figure 2**) 17, 46. By continuously releasing pro-inflammatory cytokines (such as IL-6, IL-8, TNF-α), MMPs, and growth factors into the extracellular matrix, SASP not only directly promotes the inflammatory response and fibrotic remodeling in the local tissue, but also induces “secondary senescence” in adjacent healthy cells 47-51. This spread of the pro-inflammatory microenvironment mediated by SnCs is a key mechanism driving the occurrence and development of various degenerative diseases.

### 2.3. Association between SnCs and ARDs

SnCs act as a key driver of various ARDs through their unique growth-inhibiting effect and imbalance in secretory activity 52, 53. Specifically, the irreversible growth arrest of SnCs significantly weakens the regenerative potential of damaged tissues 54. More importantly, the continuously secreted SASP (such as IL-6, IL-8, TNF-α, and MMPs) accumulates in the tissue microenvironment, inducing chronic low-grade inflammation, extracellular matrix degradation, and “secondary senescence” of adjacent healthy cells, thereby accelerating the functional deterioration of tissues 55-58. Preclinical studies have confirmed the pathogenic role of SnCs in multiple organ system disorders. In the cardiovascular system, the inflammatory response mediated by senescent endothelial cells promotes lipid deposition and plaque instability 59. In the central nervous system, senescent glial cells exacerbate neuronal damage and cognitive decline through pro-inflammatory responses 60, 61. In the respiratory and movement systems, senescent fibroblasts and chondrocytes drive the evolution of pulmonary fibrosis and osteoarthritis through promoting abnormal collagen deposition and cartilage matrix degeneration 62, 63. Furthermore, senescent renal tubular epithelial cells are the core pathological factor causing renal interstitial fibrosis in diabetic nephropathy 64.

**Figure 3:**
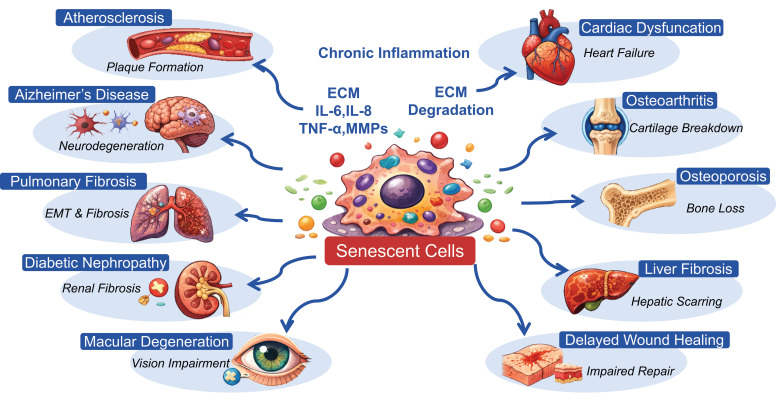
Mechanistic roles of SnCs in multisystem ARDs. SnCs disrupt tissue homeostasis through irreversible growth arrest and the pro-inflammatory effects of the SASP. SASP factors (including IL-6, TNF-α, and MMPs) drive chronic inflammation and extracellular matrix degradation, thereby promoting pathological degeneration across multiple organ systems, including the cardiovascular system (atherosclerosis and heart failure), nervous system (Alzheimer's disease), respiratory system (pulmonary fibrosis), skeletal system (osteoarthritis), and renal system (diabetic nephropathy).

Overall, the pathological aggregation of SnCs not only indicates the depletion of the body's functional reserve, but also amplifies the local cell damage into systemic organ dysfunction through the inflammatory cascade triggered by SASP. Thus, SnCs form a crucial link connecting basic biological aging and clinical pathological diseases (see **Figure 3** and **Table 1**).

## 3. Senotherapy: Intervention Strategies Targeting SnCs

Senotherapy represent a class of intervention paradigms designed to precisely modulate the fate and function of SnCs through pharmacological or bioengineering approaches 78. The core logic of this field has evolved from a singular “cell clearance” strategy into an integrated pathway of “identification—clearance—suppression—homeostatic restoration”: first, SnCs are precisely identified by exploiting their specific SCAPs, surface markers, or metabolic features 79, 80; second, apoptosis of SnCs is specifically induced through small-molecule senolytic drugs (senolytics) or immune-mediated senolytic strategies 81-83; for the remaining SnCs, senomorphic drugs (senomorphics) are applied to intervene in key signaling axes, such as NF-κB and JAK/STAT, thereby suppressing the pro-inflammatory phenotype of the senescence-associated secretory phenotype (SASP) 84, 85; finally, tissue microenvironmental homeostasis is restored through metabolic reprogramming 86. This chapter will focus on reviewing the molecular targets, pharmacological characteristics, and translational prospects of senotherapy and related technical approaches.

### 3.1. Senolytics: Cleaning SnCs

**Figure 4:**
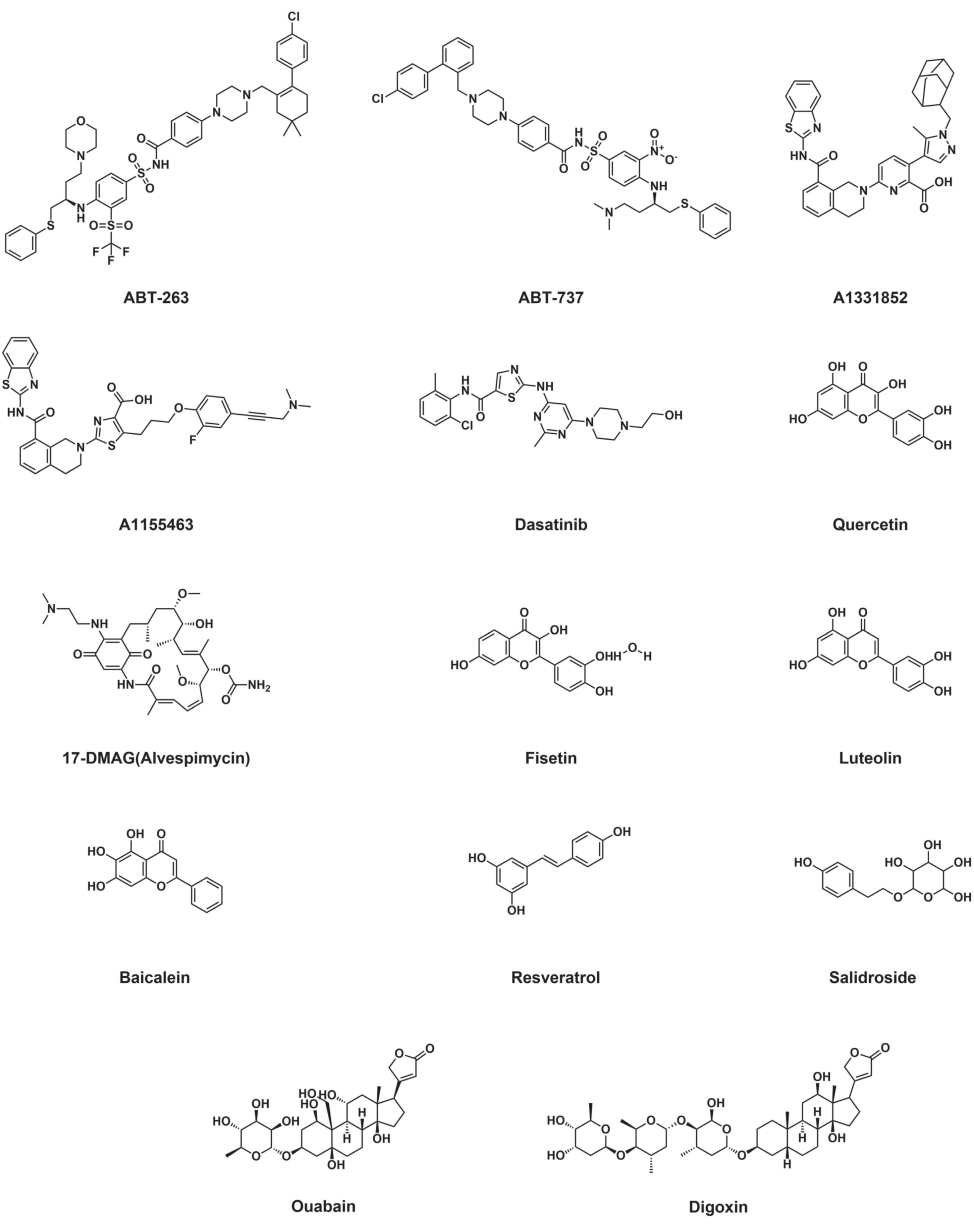
The chemical structures of representative small-molecule senolytics.

Senolytics aim to target SCAPs pathways, thereby overcoming the apoptotic resistance of SnCs and precisely inducing their programmed cell death 87. The core molecular mechanisms center on antagonizing BCL-2 family proteins, inhibiting the PI3K/AKT/mTOR cascade, or blocking specific protein-protein interactions, which subsequently trigger mitochondrial outer membrane permeabilization and the apoptotic cascade 88. This section will systematically discuss several major classes of representative senolytics, including small-molecule inhibitors and natural compounds (see **Figure 4** and **Table 2**).

#### 3.1.1. Single-target inhibitors

##### (1) BCL-2 family inhibitors

SCAPs activation is the cornerstone of SnCs survival, with differential expression of BCL-2 family members playing a particularly critical role 89-94. SnCs frequently construct a robust apoptotic defense mechanism through the specific upregulation of BCL-XL and BCL-W, thereby providing well-defined targets for the development of small-molecule inhibitors designed to disrupt this homeostasis 95, 96. First-generation broad-spectrum BCL-2 inhibitors, represented by ABT-737 and its orally bioavailable formulation ABT-263, act as BH3-mimetics; they competitively bind to the hydrophobic pocket of anti-apoptotic proteins, blocking their interaction with pro-apoptotic counterparts 94, 97. Although ABT-263 has demonstrated remarkable efficacy in clearing SnCs across multiple tissues, its clinical translation has been severely limited by dose-dependent thrombocytopenia resulting from systemic BCL-XL inhibition 98, 99.

To address this safety bottleneck, research has shifted toward highly selective BCL-XL inhibitors, such as A1331852 and A1155463. These compounds are designed to precisely eliminate BCL-XL-dependent senescent cells by triggering the caspase cascade 100. However, such high selectivity also raises the issue of a “narrow therapeutic spectrum”: the apoptotic dependencies of SnCs are highly heterogeneous, and single-target inhibition often fails to cover all pathogenic subpopulations. Future studies should focus on identifying the specific apoptotic vulnerabilities of SnCs in different tissues and on exploring novel drug delivery systems to optimize the therapeutic index.

##### (2) Kinase inhibitors

The combination of dasatinib and quercetin (the D+Q regimen) represents one of the most rapidly translated therapeutic modalities in the field of senolytics. Dasatinib, a broad-spectrum tyrosine kinase inhibitor, primarily blocks pro-survival signaling by inhibiting Src family kinases, thereby overcoming the apoptotic resistance of specific SnCs, such as preadipocytes 101.

Quercetin in this combination exerts multi-target regulatory effects. Its biological actions are highly dependent on the allosteric activation of SIRT1, which subsequently remodels cellular homeostasis through the following pathways: (a) anti-inflammatory and immunomodulatory effects: suppressing NF-κB acetylation and blocking the pathological release of TNF-α and IL-1β; (b) redox homeostasis: scavenging excessive reactive oxygen species via the SIRT1/Nrf2/HO-1 axis; (c) metabolic and apoptotic control: coordinately modulating the SIRT1/FOXO pathway and optimizing the BCL-2/BAX ratio 102, 103.

Preclinical and early clinical evidence consistently demonstrates that the D+Q regimen significantly reduces the circulating SASP profile and restores multi-system tissue function 104, 105; however, its relatively low bioavailability still needs to be optimized through novel delivery technologies.

##### (3) p53-FOXO4 axis regulator

Disrupting the physical interaction between p53 and FOXO4 is a highly efficient strategy for inducing apoptosis in SnCs. Interfering peptides represented by FOXO4-DRI can precisely overcome apoptotic resistance by releasing nuclear p53 into the cytoplasm to trigger cell death 106. In cartilage aging models, FOXO4-DRI exhibits remarkable broad-spectrum senescent cell clearance efficacy and suppresses microenvironmental deterioration; however, it also reveals a kinetic disconnect between “clearance” and “functional regeneration”, as this peptide fails to effectively induce the redifferentiation of residual cells 107.

In complex pathological settings, p53-FOXO4 interfering agents display a pronounced “double-edged sword” characteristic. In fibrotic diseases such as radiation-induced pulmonary fibrosis, these agents confer significant protection by eliminating pathological fibroblasts; however, in models such as pulmonary arterial hypertension, non-specific induction of apoptosis in critical endothelial cells paradoxically exacerbates hemodynamic disturbances 108, 109.

These contradictory outcomes underscore the functional heterogeneity of SnCs across different tissues or pathological stages, and the development of inhibitors with higher cell-type selectivity is a critical prerequisite for clinical translation.

##### (4) HSP90 inhibitor

HSP90 inhibitors precisely disrupt the anti-apoptotic signaling network essential for SnCs survival by interfering with the interaction between molecular chaperones and client proteins. The core mechanism involves inducing ubiquitin-proteasomal degradation of downstream pro-survival factors, such as AKT and ERK, thereby dismantling the apoptotic barrier 110. Recent studies have uncovered a novel role for HSP90 in regulating cell cycle checkpoints: for example, AT13387, through remodeling proteostasis, not only drives tumor cells into stable senescence but also suppresses their stemness features 111.

Moreover, the post-translational regulation of the tumor suppressor p14ARF by HSP90, mediated by CHIP-dependent lysosomal degradation, further underscores its multidimensionality as a therapeutic target 112. In senolytic screening campaigns, derivatives such as 17-DMAG have demonstrated exceptional potency. In DNA damage repair-deficient mice, targeting HSP90 systemically reduces the SnCs burden across multiple organs with nanomolar efficacy, outperforming conventional chemotherapeutic agents 113, 114. This broad-spectrum clearance capacity across diverse senescence models, including lung fibroblasts and endothelial cells, positions HSP90 inhibitors among the most promising clinical candidates.

#### 3.1.2. Multi-target inhibitors

Naturally derived small-molecule compounds have garnered considerable attention for their exceptional senolytic potential. Representative active constituents, including fisetin, luteolin, baicalein, resveratrol, and salidroside, exhibit significant clearance efficacy by differentially modulating the anti-apoptotic pathways upon which SnCs depend 115-119. The unique advantage of these phytochemicals lies in their multi-target effects: while specifically eliminating SnCs, they can synergistically remodel the immune microenvironment and initiate tissue regeneration programs, thereby offering new perspectives for the systemic intervention of ARDs.

##### (1) Fisetin

Fisetin is regarded as a senolytic agent with substantial clinical translational value. This profound clinical potential is currently being validated in several human clinical trials, highlighting its status as a premier candidate for age-related therapeutic interventions 120-123. Studies have demonstrated that fisetin selectively eliminates p16^INK4a^-positive senescent endothelial cells, and by inhibiting the release of SASP components and restoring nitric oxide bioavailability, significantly ameliorates vascular endothelial dysfunction and arterial stiffness in aged individuals 124, 125. In multiple animal models and experiments with human peripheral blood mononuclear cells, a “hit-and-run” intermittent dosing strategy not only effectively reduces the SnCs burden and circulating SASP levels, but its efficacy is even comparable to that achieved by genetic ablation approaches or synthetic senolytics 120, 126. Furthermore, fisetin downregulates the expression of aging-related genes across multiple organs, such as the brain, liver, and lung, and exerts systemic anti-aging effects by lowering plasma S100B levels 127.

##### (2) Luteolin

Luteolin exerts protective effects through a dual mechanism. First, by inhibiting stress-induced p53 phosphorylation and upregulating SIRT1 expression, it antagonizes cellular senescence and collagen degradation triggered by oxidative stress 128 or ultraviolet A radiation 129. Second, luteolin specifically disrupts the protein interaction between p16 and CDK6. In *in vivo* models, this mechanism mediates lifespan extension, amelioration of natural aging, and significant suppression of doxorubicin-induced inflammation and fibrosis 130.

##### (3) Baicalein

Baicalein exhibits distinct cell fate regulatory capabilities under different pathological contexts. For instance, in malignant tumor models such as melanoma, it induces compensatory senescence in tumor cells by inhibiting glucose uptake and blocking the mTORC1/HIF-1α pathway, thereby curbing their proliferation and migration 131. In contrast, in models of idiopathic pulmonary fibrosis, baicalein significantly attenuates the senescent phenotype of lung fibroblasts, reduces aberrant collagen deposition, and delays pathological fibrotic progression by activating SIRT3 and antagonizing the TGF-β1/Smad signaling axis 132.

##### (4) Resveratrol

Resveratrol intervenes in the progression of aging through a multidimensional signaling network. Its core mechanism involves activation of the SIRT1/AMPK axis, which significantly suppresses the accumulation of SnCs and the production of the SASP by reinforcing antioxidant defenses and anti-inflammatory responses 133, 134. Concurrently, resveratrol exhibits marked protective potential in neurodegenerative diseases, cardiac dysfunction, and metabolic disorders by optimizing mitochondrial biogenesis and modulating the apoptotic threshold 135, 136.

##### (5) Salidroside

Salidroside is regarded as a prominent nature-derived bioactive glucoside with substantial clinical translational value. This profound therapeutic potential in delaying aging and mitigating age-related pathologies highlights its status as a premier candidate for longevity interventions 137, 138. Studies have demonstrated that salidroside effectively extends lifespan and improves healthspan across multiple species by inducing a characteristic hormetic biphasic dose response, which activates intracellular adaptive pathways at low concentrations 137. At the molecular level, this longevity-promoting effect is directly driven by its capacity to bind HSP90 and inhibit its ATPase activity, thereby modulating downstream gene networks and reinforcing cellular stress resistance 138. In multiple preclinical models of brain aging and neurodegeneration, such as naturally aged mice and Alzheimer's disease models, salidroside administration significantly ameliorates cognitive dysfunction, reduces amyloid-β (Aβ) deposition, and alleviates neuronal degeneration in the hippocampal CA1 region 119, 139. These neuroprotective actions are tightly coupled with the selective downregulation of senescence-associated markers, including β-galactosidase, p21, and p16, an effect mediated by the upregulation of telomerase reverse transcriptase (TERT) expression via the PI3K/Akt signaling pathway 119. Furthermore, salidroside exerts systemic anti-aging and antioxidative effects across diverse biological systems by augmenting superoxide dismutase (SOD) activity while consistently suppressing the accumulation of malondialdehyde (MDA) and pro-inflammatory cytokines, such as IL-1β, IL-6, and TNF-α 138, 139.

#### 3.1.3. Senolytics based on homeostatic regulation

In addition to the aforementioned classical pathways, cardiac glycosides (CGs) and PPAR agonists also exhibit significant senolytic potential by modulating ion homeostasis and metabolic signaling networks.

##### (1) Cardiac glycosides (CGs)

CGs, such as digoxin and ouabain, exert their senolytic effects primarily by inhibiting the activity of the Na^+^/K^+^-ATPase (NKA) pump 140, 141. The core mechanisms are as follows: first, NKA inhibition leads to intracellular Na^+^ and Ca^2+^ overload, which subsequently activates Ca^2+^-dependent signaling such as CaMKII, triggering mitochondrial membrane potential collapse and caspase-dependent apoptosis 142; second, they suppress the secretion of SASP components by antagonizing the NF-κB signaling pathway, thereby ameliorating the chronic inflammatory microenvironment 143. Furthermore, CGs can specifically induce autophagy inhibition and apoptosis in BRAF^V600E^-positive senescent cells by modulating the Src/Akt axis 144. However, the efficacy of these agents exhibits pronounced cell-type dependence 145: studies have revealed that human mesenchymal stem cells frequently acquire enhanced K^+^ compensatory capacity and apoptotic resistance during senescence, leading to resistance to CGs.

##### (2) PPAR agonists

PPAR agonists exert anti-aging effects by precisely modulating the “metabolism-inflammation” coupling network 146-148. These agents exhibit dual molecular effects: PPAR-α activation mediates enhanced fatty acid oxidation, thereby alleviating lipotoxicity, whereas PPAR-γ activation simultaneously optimizes insulin sensitivity and synergistically suppresses pro-inflammatory responses, collectively disrupting the survival dependencies of SnCs 149, 150. At the molecular level, these agonists bidirectionally regulate BCL-2 family proteins, triggering SnCs apoptosis by downregulating anti-apoptotic factors such as BCL-XL and upregulating pro-apoptotic proteins such as BAX 151, 152. Notably, at the clinical level, PPAR-γ agonists, including rosiglitazone and pioglitazone, exhibit remarkable pleiotropic effects, encompassing the restoration of mitochondrial function and the blockade of SASP release 153. Large-scale epidemiological data further corroborate that diabetic patients receiving long-term pioglitazone treatment demonstrate significantly reduced all-cause mortality, confirming a clear association between PPAR activation, human health lifespan extension, and a diminished risk of ARDs 148.

### 3.2. Immuno-senolytics: Chasing SnCs

Immune-mediated senescent cell clearance strategies (Immuno-senolytics) represent a novel intervention paradigm that achieves the precise elimination of SnCs by activating or enhancing endogenous immune system functions 154. Distinct from conventional small-molecule Senolytics, this strategy aims to reinvigorate immune surveillance mechanisms and encompasses several innovative technological approaches. First, antibody-guided therapies target SnCs surface markers, such as urokinase-type plasminogen activator (uPAR), or block “don't eat me” signaling axes, such as CD47/SIRPα, thereby abrogating immune evasion and promoting macrophage-mediated phagocytosis 155, 156. Second, adoptive cell therapies utilize engineered effector cells, such as CAR-T or NK cells, to achieve highly efficient killing by recognizing specific antigens, including NKG2D ligands 157, 158.

Furthermore, senolytic vaccines, for instance those targeting the GPNMB protein, are capable of inducing durable adaptive immune responses to suppress SnCs accumulation over the long term 159. Concurrently, the application of immunomodulatory agents, such as cytokines (e.g., IL-15) and STING agonists, can further synergistically enhance the clearance efficiency of the immune system 160. This combined strategy, characterized by high target specificity and immune memory effects, has emerged as a highly promising frontier in the field of aging intervention. Representative agents and technical approaches are summarized in **Table 3**.

#### 3.2.1. Antibody therapy

##### (1) Cell-surface markers of SnCs

Studies have shown that uPAR is specifically and highly expressed on the surface of SnCs, where it is deeply involved in microenvironmental remodeling by regulating extracellular matrix degradation 161. Based on this phenotype, antibody-based drugs targeting uPAR can precisely eliminate SnCs by synergistically harnessing antibody-dependent cellular cytotoxicity (ADCC) and complement-dependent cytotoxicity (CDC), and their efficacy has been validated in various pathological fibrosis models 162. Furthermore, the decoy receptor 2 (DcR2), which is enriched on the surface of SnCs, is also a highly promising target: binding of a specific antibody to DcR2 can reverse the anti-apoptotic phenotype, reactivate the caspase-8-mediated extrinsic apoptotic pathway, and induce programmed cell death in SnCs 163.

##### (2) Immune checkpoints

SnCs can exploit immune checkpoint signaling to construct an immune-evasive microenvironment, and blockade of these inhibitory signaling axes can significantly enhance immune surveillance in aged individuals 164. On the one hand, the overexpression of CD47 on the surface of SnCs effectively suppresses the phagocytic activity of macrophages through “don't eat me” signaling, i.e., the CD47-SIRPα interaction. Application of anti-CD47 antibodies specifically blocks this signaling axis, generating a marked pro-phagocytic effect that demonstrates significant therapeutic potential in aging-associated tumors and fibrotic diseases 165, 166. On the other hand, reports have revealed that p16^INK4a^-positive cells evade immune recognition by upregulating PD-L1 expression, thereby accelerating the progression of pulmonary fibrosis and chronic obstructive pulmonary disease. Notably, activating PD-L1 antibodies can precisely ablate p16^+^/PD-L1^+^ SnCs via an Fcγ receptor-dependent mechanism, significantly alleviating the tissue inflammatory burden 167.

Antibody-based therapies, with their high target specificity and pharmacokinetic advantages, demonstrate potential superiority over small-molecule drugs in SnCs clearance. However, optimizing target specificity, enhancing penetration into solid tissues, and mitigating the potential risk of autoimmunity remain key challenges for translational research. Future studies should integrate single-cell sequencing technologies to identify candidate antigens and explore engineering approaches such as bispecific antibodies or antibody-drug conjugates (ADCs) to achieve more precise intervention.

#### 3.2.2. Cell therapy

Natural killer (NK) cells, as the core effector components of the innate immune system, possess the endogenous capacity to recognize and eliminate SnCs, and functionally enhancing them through genetic engineering has emerged as a key approach to improving SnCs clearance efficiency 168.

Studies have shown that SnCs can construct an immune-evasive phenotype by upregulating the expression of non-classical human leukocyte antigen HLA-E, which binds to the inhibitory receptor NKG2A on the surface of NK cells. Targeted blockade of the NKG2A/HLA-E signaling axis effectively restores NK cell cytotoxicity, indicating that this pathway represents an important target for reversing the progression of aging-related pathologies 13. Further reports have revealed that the aging process is accompanied by a characteristic remodeling of NK cell subsets, manifested by a decrease in the CD56^bright^ subset and an increase in the CD56^dim^ subset. This subset shift often leads to a decline in overall killing efficacy, thereby impairing immune surveillance and precipitating “immunosenescence” and associated degenerative diseases 168.

Currently, engineered CAR-NK cells constructed through synthetic biology exhibit more precise antigen recognition potential 169, 170. Compared with CAR-T cells, CAR-NK cells demonstrate remarkable translational value in preclinical studies of age-related malignancies and chronic degenerative diseases, owing to their lower risk of autoimmune responses, enhanced capacity for solid tissue infiltration, and unique non-MHC-restricted killing mechanism 171.

#### 3.2.3. Vaccines

Glycoprotein nonmetastatic melanoma protein B (GPNMB) is a transmembrane protein specifically upregulated on the surface of SnCs, widely distributed in tissues such as the liver, adipose tissue, and vascular endothelium, and has emerged as a highly promising molecular target in the field of aging intervention 172.

Studies have demonstrated that vaccines targeting GPNMB can effectively eliminate SnCs, markedly reverse pathological phenotypes in progeroid mice, and extend their lifespan 159. Specifically, dendritic cell vaccines loaded with cationic protein-fused GPNMB have exhibited significant efficacy in various natural aging and stress-induced aging models: by downregulating SA-β-gal activity and the expression of classical senescence signaling axes such as p16^INK4a^, p21^Cip1^, and p53, this vaccine markedly improves glucose tolerance and insulin resistance, confirming its critical role in correcting metabolic dysfunction induced by SnCs accumulation 173.

Mechanistic analyses reveal that GPNMB, as a senescence-specific antigen, elicits active immunity that specifically activates CD8^+^ T cells to precisely eliminate GPNMB^+^ senescent vascular endothelial cells and adipocytes. This process effectively curbs the progression of atherosclerosis and metabolic disorders 174.

Beyond its therapeutic targeting potential, GPNMB has demonstrated translational value as a noninvasive biomarker for monitoring SnCs burden and senotherapy efficacy *in vivo*. Notably, plasma GPNMB levels are significantly elevated in Parkinson's disease (PD) patients compared with neurologically normal controls, positively correlating with disease severity as assessed by UPDRS Part III scores across two independent cohorts; furthermore, cerebrospinal fluid GPNMB levels exhibit a protein quantitative trait locus effect consistent with the PD risk-associated rs199347 haplotype, collectively establishing blood-based GPNMB quantification as a clinically accessible readout for tracking senescence-associated pathological progression and evaluating intervention outcomes 175.

The core advantage of senolytic vaccines lies in their ability to induce durable endogenous immune memory. Compared with antibody- or cell-based therapies, the vaccine strategy holds the promise of achieving a “single vaccination, long-term protection” intervention effect and features a more favorable safety profile 176. However, this field still faces critical challenges, including the balance between antigen specificity and safety, immunogenicity optimization, and inter-individual variability.

#### 3.2.4. Regulating chemokines

CCL-2, a core component of the SASP, is markedly upregulated in various SnCs models, and its secretion levels exhibit a stepwise increase with the progression of cellular replicative senescence 177. Mechanistic studies have revealed that SnCs release substantial amounts of CCL-2 through a RAB27A-mediated lysosomal exocytosis mechanism; these pro-inflammatory factors are enriched within lysosomes and subsequently released into the circulatory system, overlapping considerably with known plasma senescence biomarkers and suggesting a pivotal role as systemic aging signals in the progression of ARDs 178.

It has been reported that in pathological models, DNA damage-induced type II alveolar epithelial cells enter senescence via a p21^WAF1/CIP1^-dependent pathway and activate the CCL-2-CCR2 signaling axis. This axis directionally recruits Ly6C^+^ inflammatory monocytes at the early stage of the lesion, and subsequently promotes excessive collagen deposition through paracrine crosstalk between activated macrophages and fibroblasts, thereby establishing a causal link between alveolar epithelial cells senescence and the initiation and progression of pulmonary fibrosis 179.

Chemokine-targeting strategies are designed to intervene in aging-associated pathologies through a dual mechanism: first, reversing the immunosuppressive microenvironment by inhibiting the recruitment of myeloid-derived suppressor cells; second, enhancing the immune surveillance capacity of NK cells and CD8^+^ T cells through the remodeling of chemotactic gradients. Although this field holds considerable promise, the construction of precise delivery systems and the optimization of multi-target combination regimens remain current bottlenecks in translational medicine.

#### 3.2.5. Other immune regulation strategies

##### (1) STING modulators

STING (stimulator of interferon genes) agonists represent important tools for modulating senescence and the associated immune microenvironment, encompassing natural cyclic dinucleotides, such as 2'3'-cGAMP, and synthetic small molecules. Their core mechanism involves inducing STING protein oligomerization or the formation of biomolecular condensates, thereby activating type I interferon signaling 180. In tumor biology, the cGAS-STING pathway has been demonstrated to induce DNA damage-associated senescence in tumor cells, suppress tumor proliferation by enhancing SASP secretion, and concurrently participate in the immune clearance of senescent cells 181, 182.

This pathway also plays a critically opposing role in certain diseases, including neurodegenerative disorders. Studies have revealed that excessive activation of cGAS within microglia triggers neurodegeneration, suggesting that this axis represents a core target for intervention in Alzheimer's disease, and the STING inhibitor H-151 can effectively alleviate neuroinflammation and cognitive impairment in the brain by suppressing microglial activation 56. Furthermore, this pathway drives cell cycle arrest by assembling an IRF3-RB complex that inhibits CDK4/6-mediated RB phosphorylation. For instance, in hepatic stellate cells, the cGAS-STING-IRF3-RB axis exerts anti-fibrotic effects by inducing their senescence 40.

However, the effects of the cGAS-STING pathway are highly context-dependent. Chemotherapy-induced tumor cells can activate cGAS signaling in polymorphonuclear myeloid-derived suppressor cells (PMN-MDSCs) via the mtDNA-VDAC channel, paradoxically enhancing tumor immunosuppression 183. This dichotomy reflects both the cellular context of cGAS-STING activation and the local immune composition. In the aged brain, cytosolic mtDNA from structurally compromised microglial mitochondria engages cGAS, triggering a type I IFN response and TNF-mediated neurotoxicity through microglial STING-TBK1 signaling, amplified by the paucity of immunosuppressive cells 56. In contrast, senescent tumor cells release mtDNA via VDAC-dependent extracellular vesicles, selectively internalized by PMN-MDSCs, where STING signals predominantly through the PERK-NF-κB axis rather than IRF3-IFN due to low intrinsic IFN production and IFNAR1 expression, thereby upregulating immunosuppressive genes such as Arg1, Nos2, and PD-L1 183. Clinically, broad STING agonism may enhance anti-tumor senescence while reinforcing myeloid immunosuppression, whereas non-selective STING blockade, though neuroprotective, may impair anti-tumor immunity. These insights highlight the need for cell-type- or compartment-specific STING modulation, such as combining senolytics to remove mtDNA-releasing tumor cells with targeted VDAC inhibition, while preserving microglial STING functions for brain homeostasis. This dual effect of “pro-senescence/anti-tumor” versus “pro-inflammation/immunosuppression” necessitates the future development of more precise tissue-specific regulatory strategies to balance anti-aging efficacy with systemic immune homeostasis 184, 185.

##### (4) TLR modulators

The Toll-like receptor (TLR) family are core sensors of innate immunity, and their modulators exhibit multidimensional potential in reshaping aging-associated immune responses. Studies have revealed that TLR signaling serves multiple biological functions in the progression of aging. First, TLR3 plays a critical role in virus-induced senescence. SARS-CoV-2 RNA, upon recognition by TLR3, not only induces cellular senescence but also amplifies the expression of the pro-inflammatory SASP, whereas TLR3 blockade can suppress this pro-inflammatory cascade 186. Second, TLR4 signaling exhibits synergistic effects in tumor senescence intervention. For example, utilizing lipid nanoparticles to co-deliver a TLR4 agonist and a STING agonist, in the context of tumor senescence induced by MEK/CDK4/6 inhibitors, effectively reverses the immunosuppressive microenvironment and activates the surveillance functions of NK cells and CD8^+^ T cells by enhancing type I interferon secretion 187. Furthermore, in intervertebral disc degeneration, TLR2/6 activation has been demonstrated to be a key factor in inducing disc cell senescence and upregulating p16^INK4a^ expression, and the antagonist o-vanillin can significantly reverse these senescent phenotypes 188.

Future TLR modulation strategies are evolving toward precision. In oncology, research is focused on enhancing tumor immunogenicity through TLR7/8 agonists and combining them with immune checkpoint inhibitors to reduce systemic toxicity. In inflammatory diseases, emphasis is placed on developing high-affinity antagonists targeting TLR7/9 to intervene in autoimmunity and COVID-19-associated cytokine storms 189-191.

### 3.3. Senomorphics: Calming SnCs

Senomorphics represent an aging intervention paradigm that is independent of eliminating SnCs, with a core mechanism centered on the precise modulation of SnCs secretory characteristics 192. By targeting key regulatory nodes such as mTOR, NF-κB, and p38 MAPK, Senomorphics can significantly suppress the expression of pro-inflammatory SASP profiles. This strategy not only curbs the pathological deterioration of the senescent microenvironment but also circumvents the potential risk of impaired tissue regeneration associated with the direct clearance of SnCs, demonstrating exceptional therapeutic potential in multiple models of chronic degenerative diseases 193, 194.

**Figure 5:**
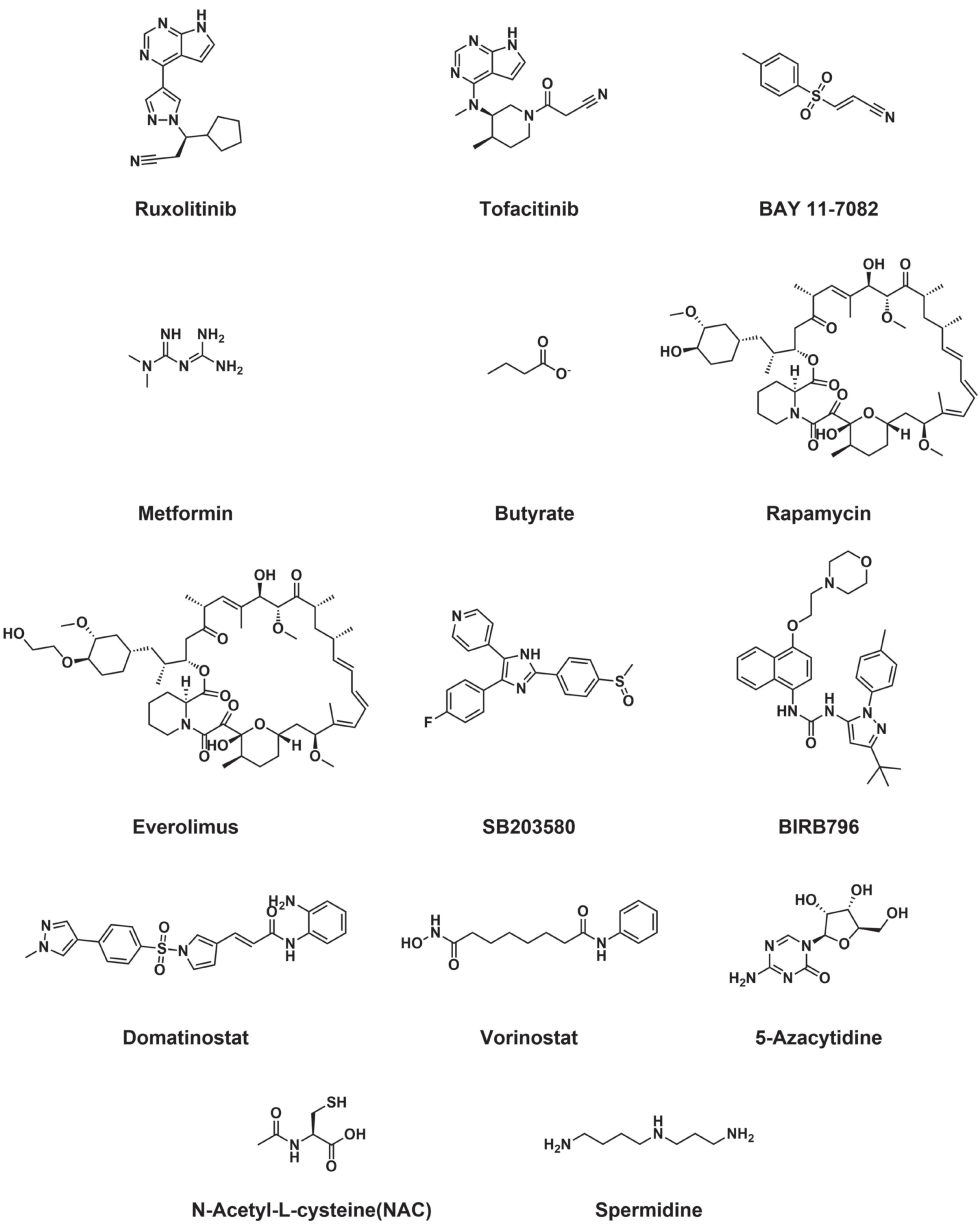
The chemical structure of typical senomorphics.

With the advancement of precision medicine, immunometabolism reprogramming based on natural polyphenolic compounds has become a research hotspot in the field of Senomorphics. By targeted modulation of cellular metabolic pathways, such agents hold promise in alleviating chronic inflammation while achieving systematic remodeling of immune functions 195. This chapter will focus on discussing typical Senomorphics, including kinase inhibitors and epigenetic modifiers, and their molecular mechanisms (see **Figure 5** and **Table 4**).

#### 3.3.1. SASP-related signaling pathway inhibitors

##### (1) JAK-STAT inhibitor

The JAK-STAT signaling pathway, an evolutionarily conserved intracellular signal transduction cascade, is composed of four Janus kinases (JAK1/2/3, TYK2) and seven signal transducers and activators of transcription (STATs), and is deeply involved in immune homeostasis regulation, inflammatory responses, and cell proliferation. JAK inhibitors specifically block cytokine-induced STAT phosphorylation and its nuclear translocation by competitively binding to the ATP-binding site of JAK proteins, thereby suppressing the transcriptional programs of downstream inflammatory genes 196, 197.

Studies have shown that STAT5 phosphorylation mediated by this pathway can upregulate anti-apoptotic factors such as BCL-2, sustaining the survival of SnCs; the application of JAK1/2 inhibitors can effectively intervene in this mechanism and promote the clearance of latently HIV-infected cells 198. In intervertebral disc degeneration models, Ruxolitinib, by blocking the JAK2/STAT3 signaling axis, significantly downregulates the expression of p16, p21, and p53 in nucleus pulposus cells and suppresses the secretion of IL-6, TNF-α, and matrix-degrading enzymes (such as MMP-3), thereby delaying degenerative progression by maintaining extracellular matrix homeostasis 199. Furthermore, Tofacitinib induces an immunosenescent phenotype in memory T cells and upregulates γH2AX expression, thereby exhibiting favorable efficacy in rheumatoid arthritis. This precise modulation of the activation and senescence status of immune effector cells provides a theoretical explanation for the balance between its clinical efficacy and the risk of infection 200.

##### (2) NF-κB inhibitor

The NF-κB signaling pathway encompasses both canonical and non-canonical activation pathways and, through the differential regulation of the IKKα/β complex, plays a pivotal role in pro-inflammatory gene expression and inflammatory homeostatic feedback 201.

Multiple lines of evidence support the value of targeting NF-κB in intervening in aging-associated pathologies. For instance, BAY 11-7082 significantly reduces the expression of pro-inflammatory SASP profiles induced by environmental stress and alleviates pulmonary epithelial cell senescence by suppressing the cGAS/STING/NF-κB cascade 202. In osteoarthritis research, dendrobine delays the switch of chondrocytes to a SASP phenotype by reducing ROS generation and inhibiting IκBα degradation and p65 nuclear translocation 203. Metformin, a clinically pleiotropic drug, has also been demonstrated to inhibit NF-κB/p65 activation induced by physical or metabolic stress, thereby suppressing the transformation propensity of SnCs by modulating the expression of MMPs and E-cadherin 204, 205.

An emerging research direction highlights the modulatory role of gut microbiota-derived metabolites in cellular senescence. Indeed, progressive alterations in the gut microbiota during chronological aging have been widely established to drive systemic frailty, orchestrating a complex, bidirectional crosstalk with host cellular senescence and immune homeostasis 206-208 Within this framework, accumulating evidence demonstrates that microbial short-chain fatty acids, particularly butyrate, exert potent senomorphic effects primarily by inhibiting the NF-κB pathway, thereby suppressing senescence in immune cells (especially T cells) and reducing SASP secretion. This gut microbiome-metabolite axis offers a novel, microecology-based therapeutic avenue for mitigating inflammaging and age-related immune dysfunction 209.

##### (3) mTOR inhibitor

mTOR inhibitors finely tune cellular metabolism and growth by differentially targeting the mTORC1 and mTORC2 complexes. Among them, rapamycin and its derivatives, as classical inhibitors, bind to FKBP12 and inhibit the FRB domain of mTORC1, thereby exerting anti-senescence effects across multiple organ systems 210.

Rapamycin has been demonstrated to improve immune responses, enhance vascular function, and downregulate skin aging markers in aged individuals; however, its benefits in the nervous and muscular systems remain controversial, and adverse effects such as hyperlipidemia and immunosuppression warrant caution 211. Everolimus, a highly selective mTORC1 inhibitor, significantly reduces SASP production by blocking the “Geroconversion” process—the transition from functional proliferation to a senescent phenotype 212. Further studies have confirmed that everolimus enhances autophagic flux by inhibiting the p70/S6K pathway, thereby protecting nucleus pulposus cells of the intervertebral disc from inflammation-induced apoptosis and offering new insights for the intervention of degenerative diseases 213.

##### (4) p38 MAPK inhibitor

The p38 MAPK inhibitors play a central role in modulating cellular stress responses and the maintenance of the SASP by blocking the mitogen-activated protein kinase pathway.

Studies have shown that SB203580 significantly suppresses the release of key SASP factors, such as IL-6, in SnCs, confirming the critical role of p38 signaling in sustaining the chronic inflammatory microenvironment 214. In conjunctivochalasis models, targeting p38 MAPK effectively reduces SA-β-Gal activity and the expression of the p53/p21 axis, thereby restoring cellular viability 215. Another potent inhibitor, BIRB 796, exhibits protective potential for the blood-brain barrier: by inhibiting oxidative stress-induced DNA damage responses and NF-κB activation, it improves the localization of tight junction proteins in endothelial cells, thus alleviating cerebrovascular dysfunction associated with neurological aging 216.

#### 3.3.2. Epigenetic modulators

Epigenetic modulators target the dysregulated epigenetic landscape in SnCs, restoring cellular homeostasis at the source of gene transcriptional regulation and thereby suppressing the expression of pro-inflammatory SASP profiles. Compared with the aforementioned strategies that intervene in downstream signaling pathways, this class of agents exhibits broader-spectrum anti-aging potential and unique therapeutic advantages.

##### (1) HDACi

Histone deacetylase inhibitors (HDACi) remodel chromatin structure by modulating histone acetylation levels, thereby regulating the expression of aging-associated genes, and exhibit a complex “context-dependent” dual mechanism of action in intervening in the pathological processes of aging 217.

On the one hand, HDACi exhibit potential senolytic effects. In melanoma models, USP7 inhibition induces SnCs to display high HDAC activity and histone hypoacetylation features; applying domatinostat, a dual HDAC/LSD1 inhibitor, can specifically eliminate such SnCs by triggering the DNA damage-apoptosis pathway, producing synergistic anti-tumor effects. This “induce-then-clear” sequential therapy offers a new paradigm for precision intervention in aging-associated pathologies 218.

On the other hand, HDACi possess protective value as a senomorphic strategy in damage models such as photoaging. Although in young cells, the broad-spectrum inhibitor vorinostat induces typical senescence phenotypes, including proliferative arrest, SA-β-gal positivity, and upregulation of p16/p21, via NF-κB activation, in UVB-induced skin photoaging models, vorinostat significantly reduces the aberrant secretion of SASP factors and MMP-1/3/9 by synergistically inhibiting the NF-κB and mTOR signaling pathways, thereby effectively attenuating tissue damage 204, 219. This functional divergence suggests that restoring the homeostatic levels of HDACs, such as HDAC7, rather than merely inhibiting them, may be key to delaying cell cycle arrest during natural aging 219.

##### (2) DNA methylation regulator

DNA methylation is a modification process mediated by DNA methyltransferases (DNMTs), involving the covalent addition of a methyl group to the 5' position of cytosine in CpG dinucleotides, and plays a central role in gene silencing and cellular identity remodeling by modulating transcription factor binding and genomic stability 220, 221.

Studies have demonstrated that the small-molecule drug TAC can upregulate telomerase reverse transcriptase (TERT) by activating the MEK/ERK/AP-1 signaling axis; TERT subsequently binds and recruits DNMT3B, inducing hypermethylation of the p16^INK4a^ promoter and thereby silencing this gene, markedly reducing the systemic SnCs burden and downregulating inflammatory cytokine levels 222. Furthermore, 5-azacytidine, a DNMT inhibitor, alleviates G2/M phase arrest by blocking DNA methyltransferase activity in models of oxidative stress-induced pancreatic β-cell senescence; moreover, it activates the eIF2α phosphorylation-autophagy pathway and nitric oxide-mediated necrotic death, thereby selectively eliminating SnCs and suppressing their pro-tumor paracrine effects 223.

In summary, epigenetic modulators target the aberrant epigenetic features of SnCs to correct cellular secretory dysregulation at the fundamental level of genetic information readout. These agents exhibit substantial clinical translational value in delaying tissue functional decline and preventing or treating ARDs.

#### 3.3.3. Other senomorphic strategies

In addition to modulating core signaling pathways and epigenetic regulation, the SASP profile can also be effectively remodeled through interventions in cellular redox balance and proteostasis.

##### (1) Antioxidant

N-acetylcysteine (NAC) is a classical thiol-based antioxidant that promotes endogenous glutathione synthesis by providing cysteine precursors. NAC effectively scavenges excessively accumulated ROS within SnCs, thereby blocking ROS-dependent activation of the NF-κB and p38 MAPK signaling axis and significantly suppressing the transcriptional expression of key SASP factors such as IL-6 and IL-8.

Studies have demonstrated that the anti-aging effects of NAC involve multidimensional mechanisms. In a bleomycin-induced lung injury model, NAC reverses the lysosomal membrane permeabilization abnormalities and autophagic flux impairment commonly observed during senescence by eliminating intracellular ROS, thereby ameliorating the pathological phenotype of SnCs and alleviating pulmonary fibrosis 224. At the clinical level, NAC significantly suppresses p16 expression in visceral adipose tissue, reduces SA-β-gal activity, and decreases the release of inflammatory markers by downregulating systemic oxidative stress, thereby improving insulin resistance while delaying obesity-associated systemic aging processes 225.

##### (2) Autophagy inducer

Autophagy is a core biological process essential for maintaining cellular proteostasis and organelle quality control. Autophagic function undergoes a progressive, stepwise decline with age, and this imbalance leads to the accumulation of damaged proteins and dysfunctional mitochondria, which in turn drives neurons and glial cells into a senescent state, thereby exacerbating the progression of Alzheimer's disease and Parkinson's disease through the release of pro-inflammatory SASP factors 226.

Spermidine, a natural polyamine that acts as an autophagy modulator, exhibits remarkable anti-aging potential. In the SAMP8 accelerated aging mouse model, spermidine enhances autophagic flux by activating the AMPK pathway, optimizes mitochondrial metabolic dynamics, and increases ATP production, thereby significantly delaying cognitive decline by attenuating oxidative stress and neuronal apoptosis 227. Studies have further demonstrated that spermidine effectively suppresses the “senescent conversion” of alveolar epithelial cells and alleviates endoplasmic reticulum stress-mediated pro-inflammatory responses by upregulating core autophagy proteins such as LC3-II, Beclin-1, and ATG7, while concurrently inhibiting the mTOR pathway 228.

Notably, in pathological studies of osteoarthritis, spermidine has been found to correct autophagic defects in senescent chondrocytes by upregulating the acetyltransferase EP300-mediated acetylation of Beclin-1 and LC3, thereby restoring the chondrogenic potential of damaged tissues 229. The above evidence indicates that targeting autophagy regulation—particularly the homeostatic remodeling achieved through spermidine—has emerged as an important strategy for intervening in multi-system aging-related diseases.

## 4. Conclusions and Prospects

**Figure 6:**
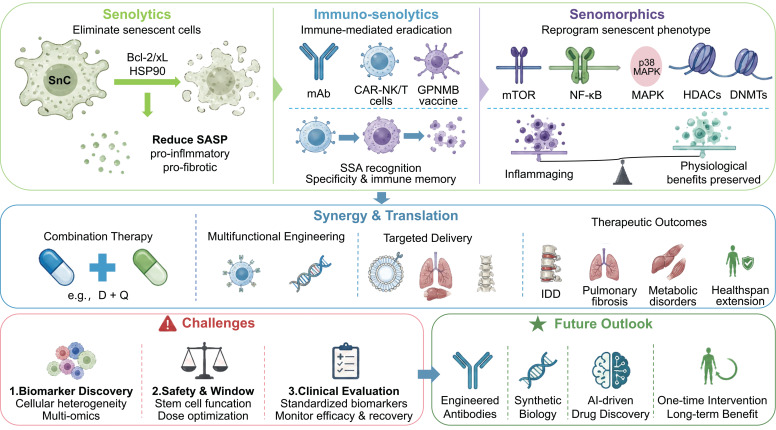
Therapeutic strategies targeting cellular senescence: from elimination to reprogramming and future translation. The schematic outlines three core modalities: Senolytics, Immuno-senolytics and Senomorphics. Central goals include reducing the pro-inflammatory, pro-fibrotic SASP, achieving specificity through SSA recognition, establishing immune memory, and preserving physiological benefits while counteracting inflammaging. The synergy & translation panel highlights combination therapy (e.g., D + Q), multifunctional engineering, and targeted delivery. Key challenges—biomarker discovery via multi-omics, safety and therapeutic window (stem cell function, dose optimization), and standardized clinical evaluation—are listed alongside a future outlook embracing engineered antibodies, synthetic biology, AI-driven drug discovery, and one-time interventions for long-term benefit.

With the deepening understanding of aging biology, intervention strategies targeting SnCs have evolved from simplistic chemical screening into a multidimensional and precise pharmacological framework. As shown in **Figure 6**, this review has systematically summarized the latest advances in both basic research and clinical translation concerning senolytics, immuno-senolytics, and senomorphics 12, 192, 230.

First, the evolution of intervention paradigms has enabled the systemic remodeling of the senescence microenvironment. By targeting pro-survival signaling pathways such as BCL-2/BCL-XL and HSP90, Senolytics selectively induce SnCs apoptosis, thereby reducing the reservoir of pro-inflammatory and pro-fibrotic SASP factors at the source 87, 88. Immuno-senolytics, including monoclonal antibodies, engineered CAR-NK/T cells, and GPNMB vaccines, precisely recognize senescence-specific antigens, offering highly specific and durable protection with endogenous immune memory for age-related diseases. Senomorphics represent a “non-ablative” intervention paradigm; by inhibiting core signaling axes such as mTOR/NF-κB/p38 MAPK or correcting epigenetic dysregulation (e.g., modulation of HDACs and DNMTs), they markedly alleviate “inflammaging” while preserving the physiological benefits of senescent cells 194.

Second, multi-target synergy and tissue-specific delivery have become pivotal for enhancing therapeutic efficacy. Evidence demonstrates that the limitations of single-target approaches are being overcome through combination regimens (e.g., the D+Q protocol) or multifunctional engineering strategies. In various models of ARDs, these strategies not only exhibit remarkable capacity for pathological reversal but also reveal the feasibility of extending healthspan via “immunometabolic reprogramming”. Emerging evidence highlights the advantages of combined or sequential senotherapeutic strategies to address the inherent heterogeneity of senescent cells (SnCs), which limits the efficacy of single-modality approaches. Combination regimens integrating senolytics (e.g., D+Q) with senomorphics or immuno-senolytics have demonstrated synergistic effects by simultaneously clearing SnCs and suppressing residual SASP, leading to superior reductions in inflammation, improved tissue repair, and reduced toxicity compared to monotherapy 231. Sequential protocols, such as pre-treatment with senomorphics followed by senolytic clearance, further minimize SASP rebound and enhance safety in models of ARDs 232.

Nevertheless, the translational leap to clinical application still faces formidable challenges 233. Future research should concentrate on the following core scientific questions:

1. Discovery of specific markers: The heterogeneity of SnCs necessitates the integration of single-cell multi-omics technologies to identify tissue- and stage-specific senescence antigens and metabolic targets 234.

2. Balancing safety and therapeutic window: Further evaluation is required to assess the potential disruption of normal stem cell function by Senolytics and to optimize long-term dosing regimens of Senomorphics to circumvent systemic toxicity. Future studies should also focus on optimizing pharmacokinetic profiles through nano-delivery systems (such as exosome- or liposome-based encapsulation 235) or chemical structural modifications (e.g., prodrug 236 and ADCs 237) to achieve more precise senescent cell intervention.

3. Construction of a clinical evaluation system: There is an urgent need to establish consensus senescence biomarkers for accurately monitoring changes in SnCs burden and the recovery of tissue physiological function following clinical interventions.

Importantly, while senotherapeutic strategies hold great promise, long-term systemic clearance of senescent cells may carry risks due to the beneficial physiological roles of transient senescent cells. Accumulating evidence indicates that senescent cells contribute positively to tissue repair and wound healing, for instance by secreting PDGF-AA to promote myofibroblast differentiation and accelerate wound closure. A classic study demonstrated that eliminating senescent cells during the acute phase of injury significantly delays tissue repair, highlighting the need to carefully balance senescent cell removal with preservation of their reparative functions to avoid compromising the body's natural damage repair capacity 238.

In summary, anti-aging drug development is at a critical juncture of transition from “broad-spectrum intervention” to “personalized precision modulation”. With the interdisciplinary integration of engineered antibodies, synthetic biology, and artificial intelligence-driven drug screening technologies, a new era of aging intervention characterized by “single intervention, long-term benefit” is rapidly approaching. Furthermore, integrating metabolic reprogramming with microecological regulation, such as exploiting gut microbiota metabolites to alleviate systemic inflammaging and immune senescence, represents a highly promising frontier for next-generation senomorphic development.

## Figures and Tables

**Table 1:** Pathophysiological mechanisms of SnCs in ARDs

Systems	Disease	Key SnCs Types	Pathophysiological Mechanisms	Ref.,
Cardiovascular Systems	Atherosclerosis	Endothelial cells, smooth muscle cells, macrophages	Activating NF-κB signaling pathway. Promoting lipid deposition and ECM degradation. Reducing plaque stability.	59
	Myocardial infarction or failure	Cardiomyocytes, cardiac fibroblasts, cardiac progenitor cells	Inducing a pro-inflammatory microenvironment and exacerbating myocardial fibrosis. Inhibit endogenous regeneration and impair contractile function.	65, 66
Nervous system	Alzheimer's	Glial cells, neurons	1.Slow down the clearance of amyloid beta protein. 2.Promote pathological aggregation of Tau protein. 3.Triggering neuroinflammation and cognitive impairment.	67
	Parkinson's	Dopaminergic neurons, PC12 cells	1.Toxic interaction between α-synuclein (α-syn-A53T) and iron overload (iron deposition) 2.SA-β-gal, p16, p21, H2A.X, γ-H2A.X, ROS accumulation, mitochondrial dysfunction 3.Dysregulation of iron-related proteins (L-ferritin, H-ferritin, DMT1, IRP1, IRP2), oxidative stress, preceding nigral dopaminergic neuron loss and motor dysfunction	68
Respiratory system	Pulmonary fibrosis	Alveolar epithelial cells, fibroblasts	Drive epithelial mesenchymal transition (EMT). Abnormal deposition of collagen. Pulmonary parenchymal remodeling.	69
	Chronic obstructive pulmonary disease	Small airway epithelial cells, fibroblasts, alveolar macrophages	Maintain chronic inflammation of the airway.	70
Urinary system	Chronic and diabetes nephropathy	Renal tubular epithelium, mesangial cells, podocytes	Triggering renal tubular atrophy and interstitial fibrosis. Disrupting the glomerular filtration barrier.	71, 72
Bone system	Osteoarthritis	Chondrocytes, synovial cells, synovial macrophages	Release MMPs to degrade cartilage matrix and induce chronic synovitis. Reconstruct the subchondral bone microenvironment.	62
	Osteoporosis	Osteoblasts, osteoclasts, and bone marrow myeloid cells	Inhibiting osteogenic differentiation and abnormally activating osteoclast activity. Disrupting bone metabolism homeostasis.	73
Digestive system	Liver fibrosis	Hepatic stellate cells and liver cells	Continuously activating the pro fibrotic microenvironment and hindering tissue functional repair.	74
Visual system	Age-related macular degeneration	Retinal pigment epithelium cells	Disrupting the integrity of the blood retinal barrier and inducing pathological choroidal neovascularization.	75
Skin system	Skin aging and wound healing disorders	Keratinocytes, melanocytes, fibroblasts	Spread aging signals. Disrupt the skin barrier. Inhibit vascular regeneration, and delay healing.	76, 77

**Table 2:** Classification, targets, and pharmacological characteristics of typical senolytics.

Category	Representative drugs/compounds	Molecular targets/mechanisms	Pharmacological characteristics	Clinical evaluation	Ref.
Single-target inhibitors	ABT-263/ABT-737	BCL-2/BCL-XL inhibitor	First generation broad-spectrum BH3 analogues	Dose-dependent thrombocytopenia	94, 97-99
	A1331852/A1155463	Highly selective BCL-XL inhibitor	Better hematological safety than ABT-263	Efficiency exhibits heterogeneity in cell types	100
	Dasatinib + Quercetin	Src / SIRT1	Synergy effect	Significantly downregulate cyclic SASP and repair multi system functionality	101-105
	FOXO4-DRI	Interference with p53-FOXO4 interaction axis	Competitive Binding of p53	Heterogeneity of effects in different pathological conditions	106-109
	17-DMAG	HSP90 inhibitor	Inducing key HSP degradation	Excellent potency (nM level) and broad-spectrum activity	110, 113, 114
Multi-target inhibitors	Fisetin	p16^INK4a^ / mTOR	Typical multi-target candidates	Exhibiting initial efficacy in reducing SASP factors and improving clinical outcomes across multiple human trials	120, 124-127
	Luteolin	p16-CDK6 / SIRT1	Antagonize stress-induced aging	Combining the potential to delay natural aging and resist fibrosis	128-130
	Baicalein	mTORC1 / Sirt3	Dual mechanism	Inhibit tumor proliferation or alleviate pulmonary fibrosis	131, 132
	Resveratrol	SIRT1 / AMPK	Strengthen antioxidant defense, optimize mitochondrial function, and inhibit SASP production	Provides protective efficacy for various ARDs	133-136
	Salidroside	PI3K/Akt/TERT pathway activator	Neuroprotective senomorphic effects; improves neuronal viability, MAP2, cognition and alleviates hippocampal degeneration.	In preclinical studies, it demonstrated a good safety profile with no obvious adverse reactions observed.	119
Homeostatic regulation	Ouabain/Digoxin	Na^+^/K^+^-ATPase (NKA)	Triggering Ca^2+^ overload mediated apoptosis	Narrow treatment window, requiring monitoring of cardiac toxicity	140-144
	Fenofibrate/Pioglitazone	PPAR agonists	Regulating the metabolic inflammatory network; Regulating the expression of BCL-2 family proteins	Long term use requires vigilance against weight gain and edema	146-153

**Table 3:** Immune-mediated senescence cell clearance strategies (Immune-senolytics)

Category	Representative drugs/technologies	Molecular targets/mechanisms	Pharmacological characteristics and clinical evaluation	Ref.
Antibody therapy	uPAR antibody	uPAR	Reshaping the fibrotic microenvironment through ADCC/ADC effect	161, 162
	DcR2 antibody	Decoy receptor 2	Reverse anti-apoptotic phenotype	163
	CD47 antibody	CD47-SIRPα axis	Block the “don't eat me” signal, enhance the phagocytic effect	164-166
	PD-L1 antibody	PD-L1	Reverse immune escape and clear SnCs	167
Cell therapy	HLA-E siRNA	HLA-E/NKG2A axis	Correction of NK cell subpopulations	13, 168
	CAR-NK cells	Targeting uPAR	High targeting with strong infiltration of solid tissues	169-171
Vaccines	GPNMB vaccine	GPNMB	Active CD8^+^ T cells and endogenous immune memory	159, 172-174
Regulating chemokines	CCL-2/CCR2 inhibitors	CCL-2-CCR2 axis	Inhibit the recruitment of Ly6C^+^ monocytes	177-179
Other immune regulation strategies	STING modulators	cGAS-STING-IRF3-RB axis	“pro-senescence/anti-tumor” and “pro-inflammation/immunosuppression”	180-183
	TLR modulators	TLR receptor	Regulating immune homeostasis through multiple signaling	186-189

**Table 4:** Summary of typical targets and mechanisms of senomorphics.

Category	Molecular targets/ mechanisms	Representative drugs	Pharmacological characteristics and clinical evaluation	Ref.
SASP-related signaling pathway inhibitors	JAK2/STAT3 axis	Ruxolitinib	Inhibit SASP secretion in nucleus pulposus cells	199
	JAK/STAT pathway	Tofacitinib	Regulating the aging phenotype and expression of γ-H2AX in memory T cells	200
	cGAS/STING/NF-κB	BAY 11-7082	Inhibit stress-induced aging of lung epithelial cells and pro-inflammatory factors	202
	NF-κB/p65 activation	Metformin	1.Regulating MMPs; 2.Inhibiting the abnormal proliferation and cancer tendency	204, 205
	NF-κB pathway	Butyrate (gut microbiota metabolite)	1.Suppresses NF-κB, immune senescence and SASP 2.Microbiota-derived senomorphic for T-cell aging	209
	mTORC1	Rapamycin	Enhance vaccine response; Improve cardiovascular function; Downregulate skin aging markers	210, 211
	mTORC1/p70/S6K pathway	Everolimus	Block the “Geroconversion” process; Protecting nucleus pulposus cells by enhancing autophagy	213, 214
	Inhibit p38 MAPK	SB203580	Downregulate the expression of p53/p21 axis; Improve conjunctival laxity	215, 216
	Synergistic blockade of p38 MAPK/NF-κB	BIRB796	Restore the localization of tight junction proteins in brain endothelial cells; Improve blood-brain barrier function.	217
Epigenetic modulators	Dual inhibition of HDAC/LSD1	Domatinostat	Induction clearance sequential mode; Synergistic USP7 inhibitor clearance of melanoma SnCs	218
	Broad spectrum inhibition of HDAC	Vorinostat	Synergistic inhibition of NF-κB/mTOR; Relieve skin photoaging damage	204, 219
	Inhibition of DNMTs enzyme activity	5-Azacytidine	Activate eIF2α-autophagy pathway; Eliminate senescent pancreatic β cells	223
Other strategies	ROS/NF-κB/p38 axis	NAC	Eliminate ROS; Reverse lysosomal membrane permeability abnormality	224, 226
	Activate AMPK while inhibiting mTOR	Spermidine	Enhance autophagy; Acetylation modification reshapes cartilage homeostasis	227-229
